# Generating Point Cloud from Measurements and Shapes Based on Convolutional Neural Network: An Application for Building 3D Human Model

**DOI:** 10.1155/2019/1353601

**Published:** 2019-09-02

**Authors:** Mau Tung Nguyen, Thanh Vu Dang, Minh Kieu Tran Thi, Pham The Bao

**Affiliations:** ^1^University of Science and Technology, School of Textile—Leather and Fashion, Ho Chi Minh City, Vietnam; ^2^Industrial University of Ho Chi Minh City, Ho Chi Minh City, Vietnam; ^3^Sai Gon University, Ho Chi Minh City, Vietnam

## Abstract

It has been widely known that 3D shape models are comprehensively parameterized using point cloud and meshes. The point cloud particularly is much simpler to handle compared with meshes, and it also contains the shape information of a 3D model. In this paper, we would like to introduce our new method to generating the 3D point cloud from a set of crucial measurements and shapes of importance positions. In order to find the correspondence between shapes and measurements, we introduced a method of representing 3D data called slice structure. A Neural Networks-based hierarchical learning model is presented to be compatible with the data representation. Primary slices are generated by matching the measurements set before the whole point cloud tuned by Convolutional Neural Network. We conducted the experiment on a 3D human dataset which contains 1706 examples. Our results demonstrate the effectiveness of the proposed framework with the average error 7.72% and fine visualization. This study indicates that paying more attention to local features is worthwhile when dealing with 3D shapes.

## 1. Introduction

A fundamental characteristic of computer-based models is the capability of describing in detail the topology and geometry structure of realistic objects. 3D modeling techniques are increasingly becoming the discipline in the computer-aided design community. In addition, many applications requiring 3D models such as human animation, garment industry, and medical research have a great impact on various aspects of human life.

Although considerable research has been devoted to practicality and visualization of 3D shapes, less attention has been paid to the problem of automatically generating a 3D model. In practice, the measurement parameters like length, perimeter, and curvature have been widely used to describe the shape of realistic objects. However, reconstructing a computer-based model from these measurements has still many gaps in approach. The major reason is that the set of sparse measurements fail to capture the complex shape variations necessary for reality. On the other hand, it is impractical to resort to scanning equipment which is time-consuming and expensive.

The aim of this study is to formulate a novel representation of a 3D model based on point cloud that would make it easy to explore the relationship between the measurements and 3D shapes using the Neural Networks system. Overall, our proposed framework creates the 3D point cloud when considering a set of measurements as input. Key to our approach is to divide an object into independent components and slices. This secession allows us to specifically define architecture of the Neural Network for each slice shape instead of working on a whole 3D shape. The point cloud not only has simple and unified textures compared to the diversities and complexities of mesh but also remains meaningful structure of object's boundaries and skeleton. Taking the 3D human model for an application, we here demonstrate an end-to-end procedure of synthesizing a new human model given anthropometric measurements and a set of parameters learned from training data.

## 2. Related Works

One of the first attempts to solve for 3D model reconstruction problem was template model based. More precisely, this method produces a new model by deforming a template model. Allen at el formulated an optimization problem to find an affine transformation at each vertex of the designed template model for fitting a 3D scanned human body. They defined three types of error and combined them to create the objective function. Their approach also dealt with incomplete surface data and filled in missing and poorly captured areas caused by the scanner [1]. Modifying the method of Allen, Hasler performed nonrigid registration with the aim of fitting pose and shape of 3D scans form a template model [2]. Seo and Magenat deformed an existing model to obtain the new one based on two stages preprocessing: The skeleton fitting found the skeleton structure that approximates the corresponding 3D human body. The skin fitting calculated the displacement vector of each vertex between the template model after skeletal fitting and the scan mesh fitting [3].

The other approach is 2D-based reconstruction. This method reduces the cost because it only requires a set of images. However, the image data often contain noises and background which are hard to remove. Blanz's approach took a human face color image as an input and generated the corresponding 3D face model. New faces and expression could be described by forming linear combinations of prototypes [4]. In their work, the weight vector was assumed to distribute as multivariate Gaussian and could be found by maximum posterior probability. Chen attempted to automatically reconstruct more complex 3D shapes like human bodies from 2D silhouettes with the shape prior which was learned directly from existing 3D models under a framework based on GPLVM [5]. However, this approach is not realistic because relying on the silhouettes only will cause the loss of depth information of a human body.

Most of the solutions come from the statistics-based approach. Similar to our approach, these methods use the training set to learn the correlation between input and output, or construct an example space for extrapolation. Inspiring form DeCarlo et al.'s work [6], the statistics-based model has become a powerful tool for demonstrating the feature space of the 3D model. In their study, human face measurements were used to generate 3D face shapes by variational modeling while a prototype shape was considered as a reference. Allen reduced the dimension of 3D human meshes from 180,000 elements to 40 or fewer by using principal component analysis (PCA). Then, linear regression was used as a technique to find the relationship between six different anthropometrics and 3D human model [7]. Seo defined two synthesizers which were joint synthesizer and displacement synthesizer. Joint synthesizer handles each degree of freedom of the joints; in other words, this synthesizer constructs the skeleton for the model, while another synthesizer was used to find the appropriate displacement on the template skin. These synthesizers were all learned from eight body measurements with the corresponding model by the use of Gaussian radial basis functions [8]. With the same approach to Allen's research, Chu et al. attached a procedure of feasibility check to determine whether the semantic parameter values input by the user is rational. The feasibility check was based on the mathematic concept of the convex hull, and if the input parameters failed the check, their system would return the most similar model in the training data [9]. Wang analyzed a human body from laser-scanned 3D unorganized points through many steps [10]. He built the feature wireframe on the cloud points by finding the key points and linking all of them with curve interpolation. After that, feature patches were generated by using the Gregory patch and updated by a voxel-based algorithm. According to the introduced feature model, anthropometric measurements are easily extracted so that he used numerical optimization to generate a new 3D human body which is extracted measurements are likely to the user input sizes. Baek and Lee performed PCA on both the body size and body shape vectors; then they found the weight values of the new model based on the parameter optimization problem with the constraints were the 25 user input measurements [11]. They also clustered hierarchically their shape vector space by an agglomerative cluster tree to remain small variation in each cluster. Wuhrer and Shu introduced a technique that extrapolates the statistically inferred shape to fit the measurement data using nonlinear optimization [12]. First, PCA is applied to produce a human shape feature space; then shape refinement is used to refine the predicted model. The objective function is formulated based on the sum of square error of three types of measurements. The author announced that the method could generate human-like 3D models with a smaller training dataset. The above methods have been suffered from a common drawback, which is the limitation of generated shapes to the space spanned by the training data. In other words, finding a large number of variables by optimizing on the small dataset would lead to the underfitting problem.

## 3. Methodology

In this section, we demonstrate our method which consists of two main steps: generating primary slices and refining 3D point cloud. 3D objects are formed by a set of planes which are perpendicular to the axial height of the object. In other words, building 3D shapes is equivalent to building all these planes. Normally, if the surfaces are smoothly divided (the distance between two adjacent planes is very small), adjacent surfaces will have nearly similar shapes. Moreover, not all measurements are available in practice; thus, we only considered some available ones as the measurements corresponding with primary planes. Therefore, selecting the main planes helps us to reduce the number of calculations and also necessary measurements.

Let us assume that the set of all surfaces that are perpendicular to the axial height of a 3D object is *S*={*S*_*i*_| *i*=1,…, *m*}. The primary set is a subset of *S*, *PS*={*S*_*i*_ ∈ *S*| *i* ∈ *PI* ⊂ {1,2,…, *m*}} such that for all *S*_*i*_, *S*_*j*_ ∈ *PS*,  *i* ≠ *j*, *S*_*i*_ and *S*_*j*_ do not have a common shape. We assessed the degree of differences of two shapes based on observing the 3D object structure. To learn the relationship between measurements and each primary surface, we construct a map from an initial set to a target set:

(1) $$\begin{array}{c} f_{i}:C_{i}=\left\{\left(x,y\right)\in {\mathbb{R}}^{2}\;\left|x^{2}+y^{2}\leq {\left(\frac{m_{i}}{2\pi }\right)}^{2}\right.\;\right\}⟶S_{i}^{\prime }, \end{array}$$

such that the difference of *S*_*i*_ and *S*_*i*_′ is smallest. If we consider hollow 3D objects and the surfaces turn into the slices defined in the following section, *C* will be a circle with its radius is computed by the perimeter of the corresponding slice.

From the principal surfaces, we can interpolate the whole 3D object since the surfaces between two principal slices whose shape gradually changing to match the shape of these two principal slices. However, the interpolated surfaces are not as practical as the actual ones. We overcame this problem by using the adjusting model that will be clarified in the next section.

### 3.1. Building Primary Slices

**Figure 1:**
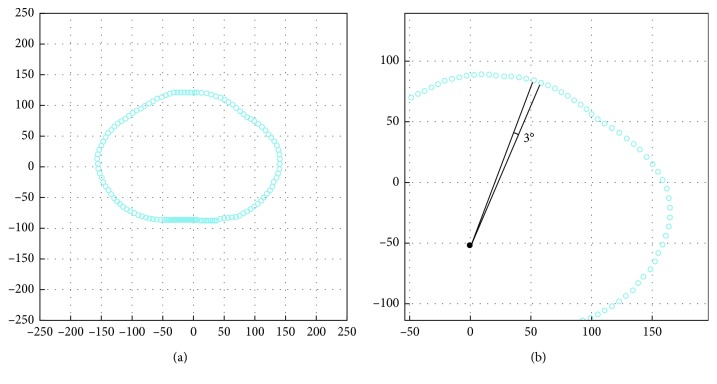
Example for our defined slice, each point is 3° apart.

We restricted our study to a class of surfaces which can be written under the trigonometric formula. Represent a surface of 3D point cloud by a set of points *S*_*z*_*o*__={(*x*, *y*, *z*) ∈ *ℝ* ^3^|*z*=*z*_0_}, so that for all *θ* ∈ [0,2*π*] and *r* > 0, there is no more than one point (*x*, *y*, *z*) ∈ *S*_*z*_ which satisfied

(2) $$\begin{array}{c} \left\{\begin{array}{c} x=x_{0}+r\;\cos\;\theta ,\\ y=y_{0}+r\;\sin\;\theta , \end{array}\right. \end{array}$$

where (*x*_0_, *y*_0_) is former given, in this study, we called it as “anchor point” which is the center of a slice (Figure 1). We named the data structure defined above as “slice structure.”

The above surface description has an advantage that the redundancy of the third dimension is eliminated. A point (*x*, *y*) could be replaced by a pair (*r*, *θ*), but the *θ* variables are actually in common for all slices. Thereby, a slice is written as a vector of the distances between the anchor point and points on this. Moreover, this representation is invariant under translation because of the equability of *r* when we translate 3D models. The rotation is also easy to handle since we merely shift the components of slice vectors.

Let *PI* ⊂ {1,2,…, *m*} is an index set of main slices, we approximated the target slices *S*_*i*_′,  *i* ∈ *PI* by formula (3). Let *S*_*i*_, *i*=1,…, *m* is the *n*-dimensional vector representing the *i* slice, the *k* ^th^ component of it is the distance between the center, and the point at *θ*=2*πk* − 1/*n*,  *k*=1,…, *n*. We defined the deformation function *f*_*i*_ : *ℤ* ^+*n*×1^⟶ *ℤ* ^+*n*×1^ as

(3) $$\begin{array}{c} S_{i}^{\prime }=f_{i}\left(X_{i}\right)={\left(W^{2}\right)}^{\text{T}}\alpha \left({\left(W^{1}\right)}^{\text{T}}X_{i}\right),\; \end{array}$$

where *W* ^1^ ∈ *ℝ* ^*n*×*L*_1_^,  *W* ^2^ ∈ *ℝ* ^*L*_1_×*n*^, *α* is a nonlinear function, *X*_*i*_ is an initial slice, and *f*_*i*_ is also called as the Multilayer Neural Network (MNN) model.

Algorithm 1 summarizes the learning procedure of generating principal slices.

**Figure 2:**
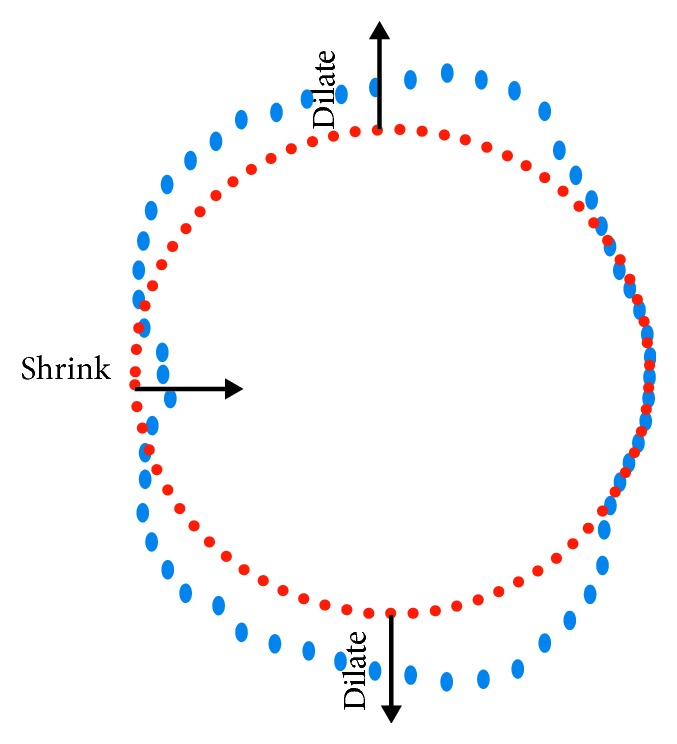
Deforming an initial slice (red circle) into the target slice (blue curve).

The key idea of the first model is to deform an initial shape into the desired shape controlled by the perimeters and the training data. Object circumferences are only useful when the object shape is revealed; hence, using circumferences alone to construct an object in detail is insufficient. Therefore, our approach also based on the shape of objects that can be extracted by NN model from the training set. In this work, the learning model seeks for positions on the initial slice that needs to be shrunk or dilated (Figure 2).

### 3.2. Generating Point Cloud

**Figure 3:**
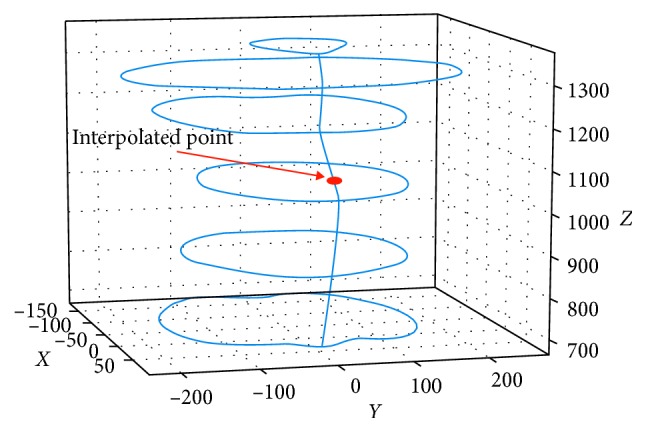
Interpolation of intermediate slices.

Based on the results of the above step, we performed interpolation on all the remaining slices. In more detail, considering *θ*=2*π*(*k* − 1)/*n*, we calculated *s*_*ik*_′,  *i* ∉ *SP* based on *s*_*ik*_′,  *i* ∈ *SP*, and this simple task was done by linear interpolation (Figure 3). We used these interpolated slices as the input for the second model. We constructed the second synthesizer based on the Convolutional Neural Network (CNN) [13] because its kernels have an ability to capture local characteristics and that is especially useful when we have to take the relationship of adjacent slices into account. This model corrected wrong interpolated points by using information on the training set via CNN architecture. The local structure of 3D shapes was retained by convolutional layers in CNN, hence resulting in fine refinement. We defined our CNN model as a function *g* : *ℝ* ^+*m*×*n*^⟶ *ℝ* ^+*m*×*n*^:

(4) $$\begin{array}{c} Y=\;g\left(X\right)=W^{L}*\alpha_{L-1}\left(W^{L-1}*\alpha_{L-2}\left(\cdots \alpha_{3}\left(W^{3}*X\right)\right)\right), \end{array}$$

where *a*_*l*_, *l*=3,…,  *L* − 1 is a nonlinear activation function and *X* is formed by stacking both principal and interpolated slices in rows.

A rational choice of loss function for this problem is Mean Square Error (MSE). In this study, MSE calculated the difference between the generated and the actual value of each point distance on each slice. We used this metric to evaluate the error on both learning models (Algorithms 1 and 2). We also added the error term of perimeter into the first model's loss function.

## 4. An Application for Building 3D Human Model

### 4.1. Dataset

The datasets used in this work were independently developed by two universities in Vietnam Table 1 summarizes our datasets (Table 1).

Each sample on both datasets was generated by the 3D scanning device and saved under “.obj” format. Each person only provided one 3D scan of the body; hence, the number of participants and samples is equal. Participants were suggested wearing a tight suit and complied with the standard pose when scanning their body. We split a 3D avatar into five parts: are torso, left leg, right leg, left arm, and right arm 

**Figure 4:**
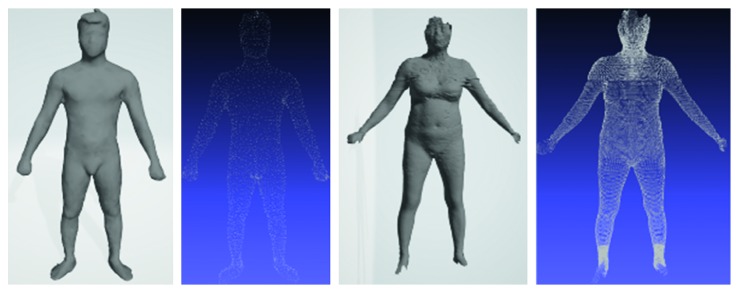
Avatars in male and female dataset and its point cloud.

**Figure 5:**
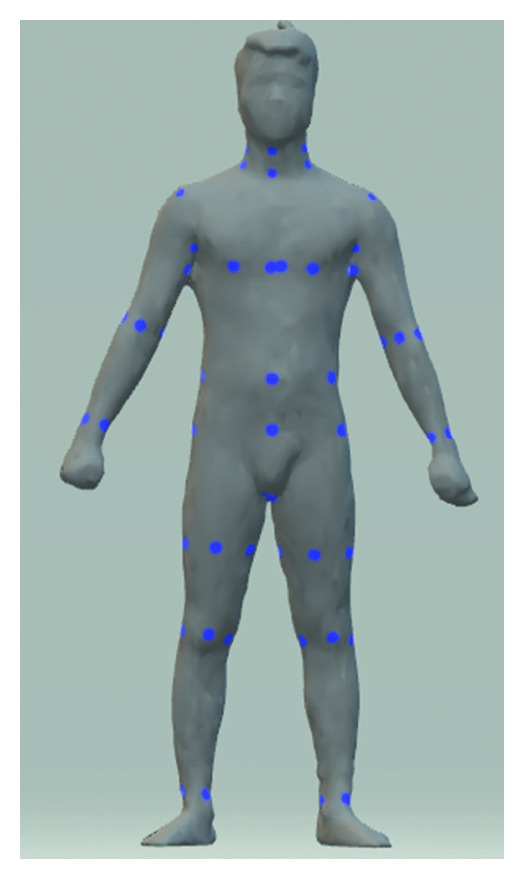
Some anatomical landmarks of a male model.

In detail, these datasets were built from different devices; thus, they have some distinct features (Figure 4). The most noticeable thing is that the point density of the male avatars is not as dense as that of females. 3D female avatars have unified structure and each their vertex was distributed into one of five above parts. Each point on torso slices, leg slices, arm slices is 3, 5, 10 degrees, respectively, apart. In addition, all slices are equally spaced by the same distance in height. Meanwhile, the male dataset did not meet the ideal condition like its counterpart. Not only it has no predefined boundary between two parts but also the point cloud does not follow our slice-structure. For this reason, the creator of the male dataset provided a set of landmarks for each avatar, and we used them as reference points to perform partition on the man model (Figure 5). Moreover, our slice-structure could be achieved by proper preprocessing steps.

### 4.2. Preprocessing

**Figure 6:**
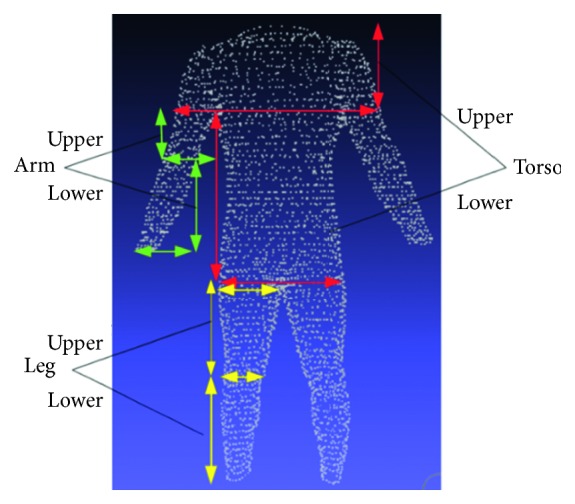
3D human model partition.

We split the whole 3D human model into five parts (Figure 6) as the following manner, and the positions mentioned below are in the landmarks set.

- Torso
  - Upper torso: From neck to armpit, limited by left elbow and right elbow.
  - Lower torso: From armpit to hip, limited by left and right hip or limited by left and right stomach.
- Arm (Left/Right)
  - Upper arm: From armpit to elbow, limited by armpit and elbow.
  - Lower arm: From elbow to wrist, limited by elbow and wrist.
- Leg (Left/Right)
  - Upper leg: From hip to knee, limited by crotch point and knee.
  - Upper leg: From knee to ankle, limited by knee and ankle.

After determining all parts of a human model, we made dividing slices based on planes perpendicular to the high axis. Let us assume that the set of all points containing in a human part is *S*, we assigned

(5) $$\begin{array}{c} \left(x_{0},y_{0},z_{0}\right)≔\left(x_{0},y_{0},\min H_{z}+i\frac{d_{z}}{m-1}\right). \end{array}$$

**Figure 7:**
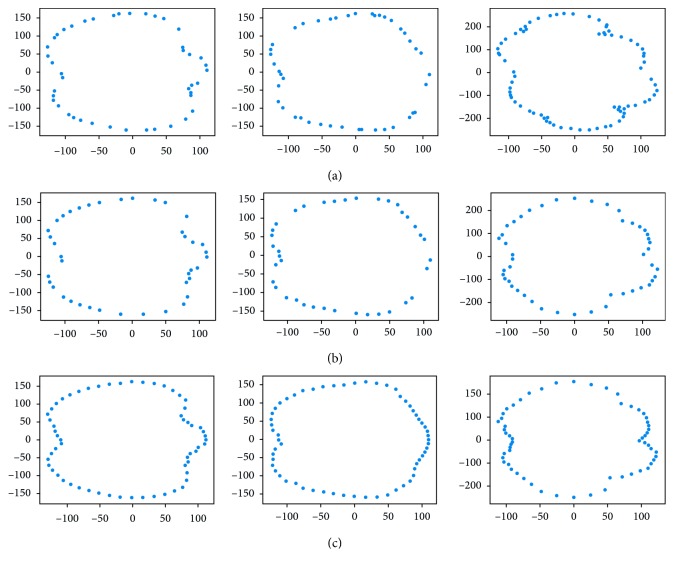
Preprocessing step. (a) Original slices. (b) After selecting slices components. (c) After filling missing data.

If

(6) $$\begin{array}{c} \min H_{z}+i\frac{d_{z}}{m-1\;}\leq z_{o}<\min H_{z}+\left(i+1\right)\frac{d_{z}}{m-1}, \end{array}$$

where *H*_*z*_={*z* ∈ *ℝ*, (*x*, *y*, *z*) ∈ *S*},  *d*_*z*_=max*H*_*z*_ − min*H*_*z*_,  *i*=0,…, *m* − 1  and *m* is the number of slices (50 in our experiment), *S*_*i*_={(*x*_0_,  *y*_0_, *z*_0_)|*z*_0_=min*H*_*z*_+*id*_*z*_/*m* − 1} (Figure 7(a)).

The next step is to construct the slice vectors. First, we calculated the position of an anchor point on each slice. The mean formula is suitable to find these points:

(7) $$\begin{array}{c} \left(x^{\left(i\right)},y^{\left(i\right)},z^{\left(i\right)}\;\right)=\frac{1}{\left|S_{i}\right|}\underset{\left(x,y,z\right)\in S_{i}}{\sum }\left(x,y,z\right). \end{array}$$

**Figure 8:**
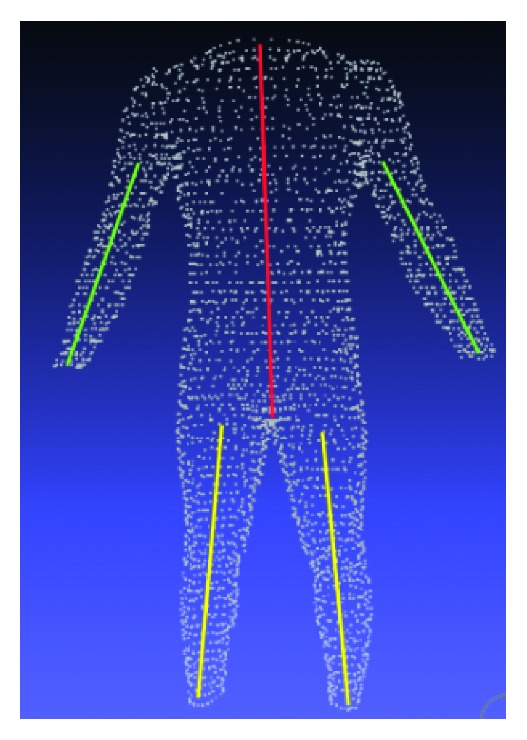
Anchor lines of a human model.

However, there are some downsides on the approach discussed above. Firstly, there are some slices that the count of points on them is not sufficient enough to approximate the actual center point. The second thing is when constructing a new human model, we need a skeleton of it. In other words, it requires an available set of anchor points. Thanks to the landmark set, we could approximate the skeleton of male avatars. Take the torso, for example, we constituted its skeleton by the line connecting the center of four neck landmarks and the crotch point (Figure 8). Once the anchor lines were found, calculating the anchor points at any height is a trivial task. The template skeleton was formed based on analyzing the position of all anchor points on the whole training dataset. In our work, we simply built the skeleton template by taking the average of the anchor points of each slice.

Given (*x*_0_, *y*_0_, *z*_0_) ∈ *S*_*i*_, the angle established by the anchor point (*x* ^(*i*)^, *y* ^(*i*)^,  *z* ^(*i*)^) and this point is computed by

(8) $$\begin{array}{c} a_{0}=\arctan\left(\frac{y_{0}-y^{\left(i\right)}}{x_{0}-x^{\left(i\right)}}\right). \end{array}$$

The *j* ^th^ component of a slice vector represents the distance between the anchor point and the point at *θ*=2*πj* − 1/*n*, and *n* is the dimension of the slice vectors. One point is distributed to the *j* ^th^ position if the following condition is satisfied:

(9) $$\begin{array}{c} 2\pi \frac{j-1}{n}\leq a_{0}<2\pi \frac{j}{n}, \end{array}$$

where *j*=1,…, *n*. The distance is directly calculated by Euclid metric:

(10) $$\begin{array}{c} d_{j}^{\left(i\right)}=\sqrt{{\left(x_{0}-x^{\left(i\right)}\right)}^{2}+{\left(y_{0}-y^{\left(i\right)}\right)}^{2}}. \end{array}$$

Both male and female avatars suffer from missing data problem. In the female dataset, the reasons are the carelessness during the scanning process and the outdated equipment. On the other hand, the missing value issue on preprocessed male models is inevitable because their original point cloud is not ideal. Furthermore, the point density is not sufficiently dense to divide the male body into many slices. We tackled this problem by performing linear interpolation on grid data of slice vectors (Figure 7(b)).

### 4.3. Measurements

**Figure 9:**
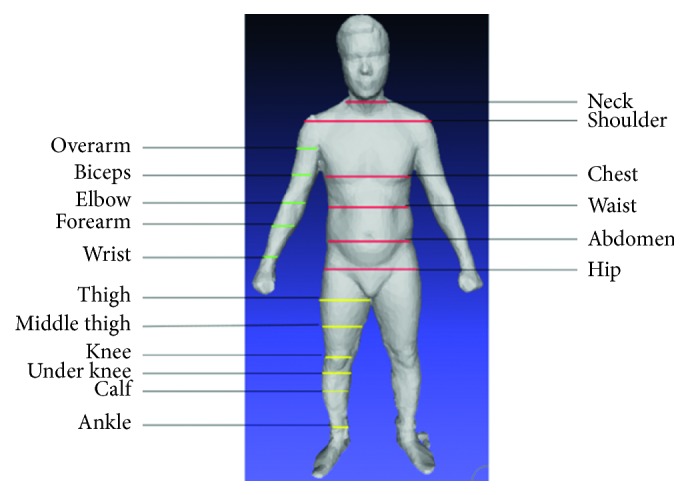
Position of primary slices.

The male dataset supplied us a set of anthropometric measurements with 178 categories comprising slice perimeters, width, and height of body parts. Nevertheless, the measurements are not in the same unit with distances computed on the point cloud. Meanwhile, the female dataset provided no measurements. Due to these reasons, we decided to recalculate the measurements to be consistent in both datasets. The simple way to compute a slice circumference is summing all distances of two adjacent points, but it does not seem realistic when measuring nonconvex shapes. We proposed using the circumference of the convex hull of a slice for its measurements. These sizes were calculated on the primary slices (Figure 9).

In summary, there are 28 slice measurements, but we can reduce the number of measures to 17 because of the similarity of the right and left sides. In addition, it is necessary to record the height (length) of each body to entirely build up the 3D human model. This would lead to 20 measurements in total. The primary positions were chosen based on the statistics on the dataset and the standard ratio of the human body [14].

### 4.4. Learning Model

**Figure 10:**
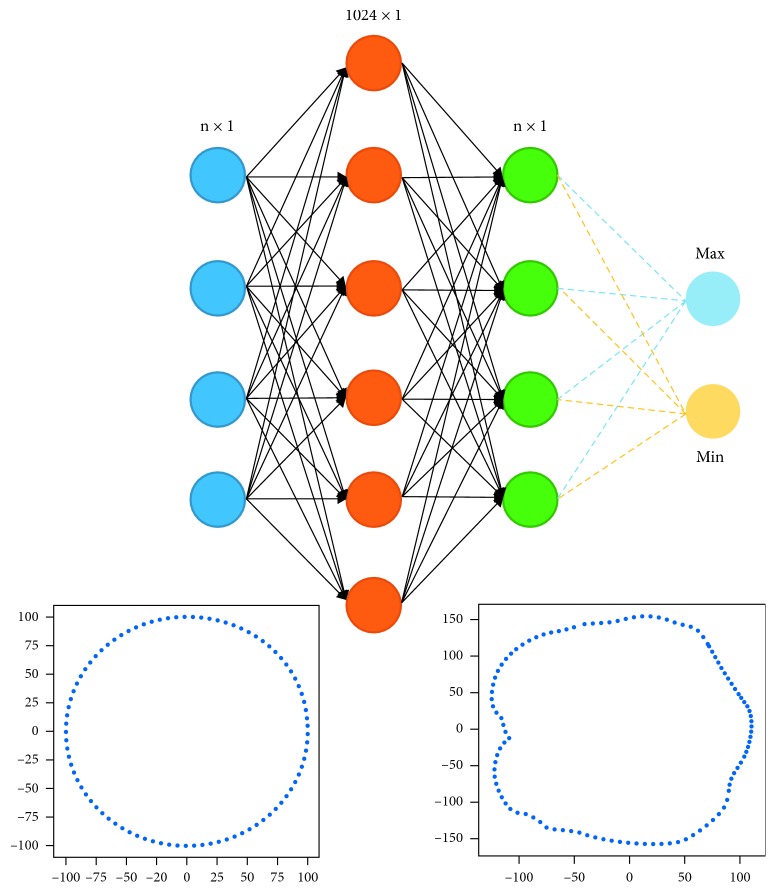
Neural Network model for generating the primary slices.

To construct primary slides, we built Neural Network (NN) models with one hidden layer as described in Section 3.1. These models deformed input slices into target slices (Figure 10).

These models take an initial circle as input and learn the deformation from the input shape into the target shape. The radius of the initial circle is *r*=*p*/2*π*, where *p* is the perimeter of the slice being considered. The input and output size depends on the body part, in our experiment, *n* equals 20, 30, and 60 for the arm, leg, and torso, respectively. The error between a predicted slice and the actual slice is calculated by

(11) $$\begin{array}{c} L\left(y,\;y^{\prime }\right)=\frac{1}{n}\overset{n}{\underset{i=1}{\sum }}{\left(y_{i}-y_{i}^{\prime }\right)}^{2}+\gamma \left(2\pi \sqrt{\frac{{\left(\max y^{\prime }\right)}^{2}+{\left(\min y^{\prime }\right)}^{2}}{2}}-p\right), \end{array}$$

where the second error term comes from the difference between the approximation of the circumference of predicted slice and the actual one. The objective function reflects the error not only at each component (local information) bust also the perimeter of the slice (global information).

**Figure 11:**
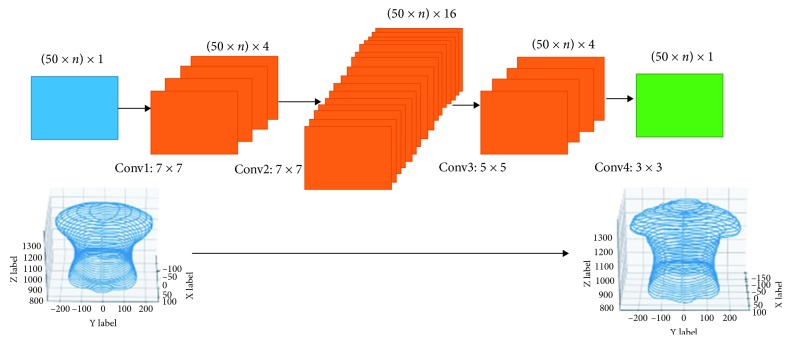
Neural Network model for adjusting entire slices.

Once the entire main slices had been found, linear interpolation was used to infer all remaining slices. These interpolated slices are the input for the second NN model as described in Section 3.2 (Figure 11). We used ReLU [15] as the activation function in both architectures.

**Figure 12:**
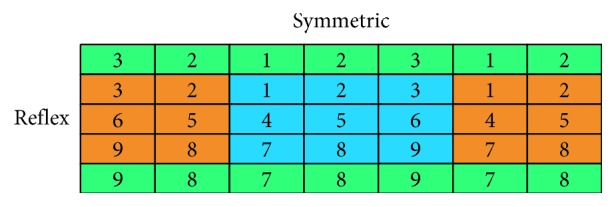
Padding strategy.

The convolutional layers help the model to learn the local correlation of adjacent slices. As a result, the remaining slices would be corrected based on the primary slices. The most cautious thing when building CNN for this problem is padding. To retain the size of data when transferring through multiple layers, we performed reflex padding in vertical and symmetric padding in horizontal (Figure 12). Padding this way retains circularly linked characteristic of slices.

We defined the loss function on this second model by using MSE:

(12) $$\begin{array}{c} L\left(y^{\prime },y\right)=\frac{1}{mn}\overset{m}{\underset{i=1}{\sum }}\overset{n}{\underset{j=1}{\sum }}{\left(y_{ij}-y_{ij}^{\prime }\right)}^{2}, \end{array}$$

where *y*, *y*′ are, respectively, a matrix of the actual and predicted distances to the anchor point of a body part.

## 5. Experiments and Results

We trained our NN models on the Linux server with 24 GB RAM, GPU with 12 GB RAM, and Xeon CPU with 2.2 GHz. We used Python as the implementing language and the main libraries using in our experiment are pytorch and numpy. We used Adam algorithm [16] to minimize the objective function, the meta parameters were set according to recommendation of the authors (learning rate *α*=0.001, *β*_1_=0.9, *β*_2_=0.999). We evaluated the error by the average relative error:

(13) $$\begin{array}{c} L\left(y,y^{\prime }\right)=\frac{1}{mn}\overset{n}{\underset{i=1}{\sum }}\overset{m}{\underset{j=1}{\sum }}\frac{\left|y_{ij}-y_{ij}^{\prime }\right|}{y_{ij}}\times 100\%, \end{array}$$

where *y*, *y*′ are, respectively, a matrix of the actual and predicted distances to the anchor point of a body part. The above error formula is not affected by the heterogeneity in size on different body parts and also on different datasets. In the male dataset, we used 1066 samples as training data and 100 samples as testing data, while 500 and 100 as training and testing data in the female dataset, the samples were selected randomly.  Table 2 shows the average error on each primary slice after training 1000 epochs on the male and female datasets.

**Figure 13:**
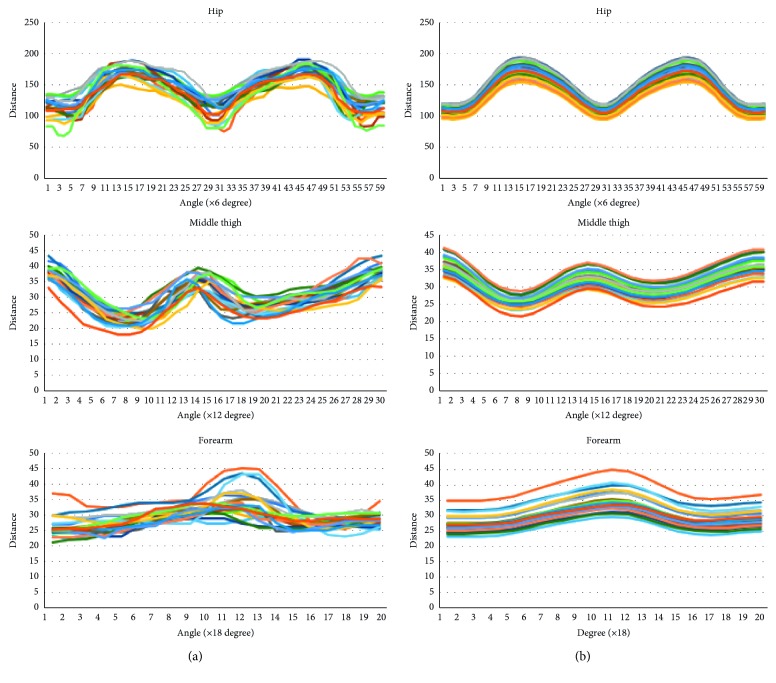
Slice vector “curves” of wrist, hip, and thigh of 20 examples in male dataset.

Learning the relationship between the size and corresponding slice shape is a hard problem because of the curse of dimension. Despite the fact that the input is just scalar, we have to predict the slice vector with at least 20 components. To solve this problem, we used initial shapes. The initial shape not only is a rough approximation for the target slice but also helps the NN model increase the number of parameters and avoid underfitting. In our work, we limited the class of initial shapes to circles that their radius is calculated by the slice perimeters. Geometrically, the first NN models act as a figure deformation controlled by the slice sizes. The NN models are the nonlinear transformations from straight lines to the particular “slice vector curves” which are the slice shapes after converting into the slice vector representation. These curves have analogous shapes if they are placed at the same position (Figure 13).

**Figure 14:**
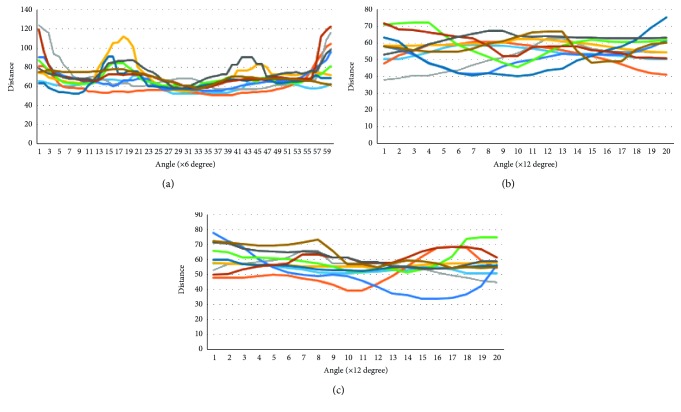
Slice vector “curves” of neck, left, and right overarm of 10 examples in the male dataset.

In the torso part, the neck slices have the highest average error because these slices are not clearly separated from the head, and the anatomical landmarks at neck position are placed at the wrong locations like collar or chin. This reason leads to that the shape of the neck slices varies considerably. The same thing happens with overarm slices. The boundaries between arms and shoulder are not accurately determined based on the landmarks. Another problem is the lack of a large number of components on overarm slice vectors because of the obstructed locations such as armpits which are ignored by the 3D scanner (Figure 14).

Table 3 shows the result after training the CNN models to entirely construct a full human body. To conduct this section, we also used Adam algorithm with 1000 epochs. We chose 50 good samples and 50 damaged samples to form the test set. Thus, we could evaluate the influence of bad patters on the overall test accuracy. The results show that the errors in the undamaged test sets are approximate to the training errors. On the other hand, the errors in the damaged test sets are not as low as the good ones. Based on the results, we can conclude that our framework is nonsensitive to the small amount of damaged samples. Moreover, the amount of samples in the training set is sufficient to do inference on the shape of testing samples.

**Figure 15:**
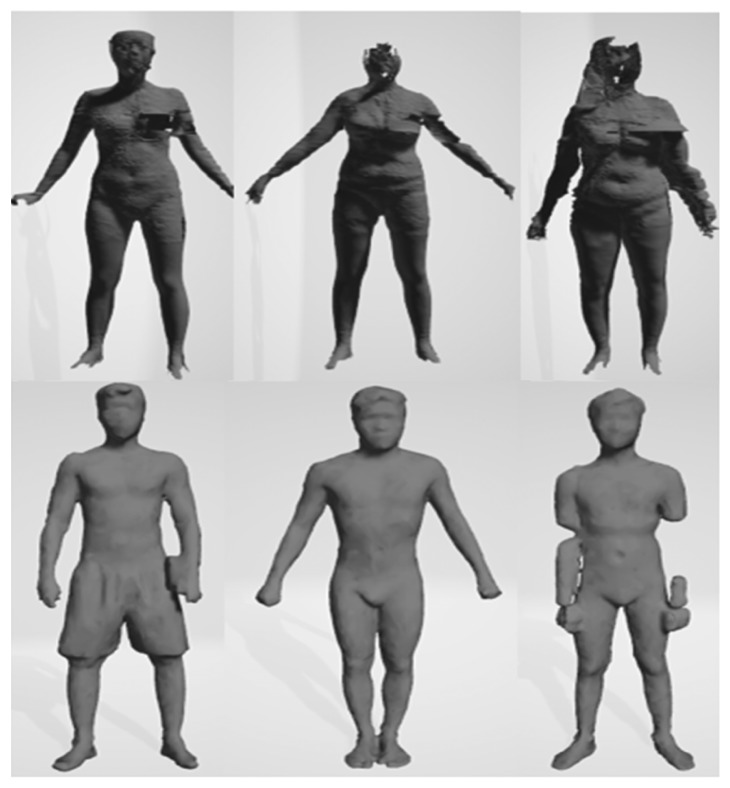
Examples for the damaged 3D human model.

While analyzing the database, we realized that there are many damaged samples in both datasets. The issues in the female dataset almost come from the scanning device, while the problems in the male dataset are due to the noncooperation of participants (Figure 15). We eliminated all unqualified samples from both datasets. Overall, there are 65 samples in the male dataset and 63 samples in the female dataset. After removing these patterns, we conducted a new training procedure on the new training and testing sets, and the results are shown in Table 4. In the male dataset, there are 1001 training samples and 100 testing samples, while there are 437 and 100 samples as training and testing data in the female dataset. The average errors after feeding the interpolated primary slices into the CNN models are lower than their own errors when compared to the ground truth.

The average training and testing time per body part are shown in Table 5.

**Figure 16:**
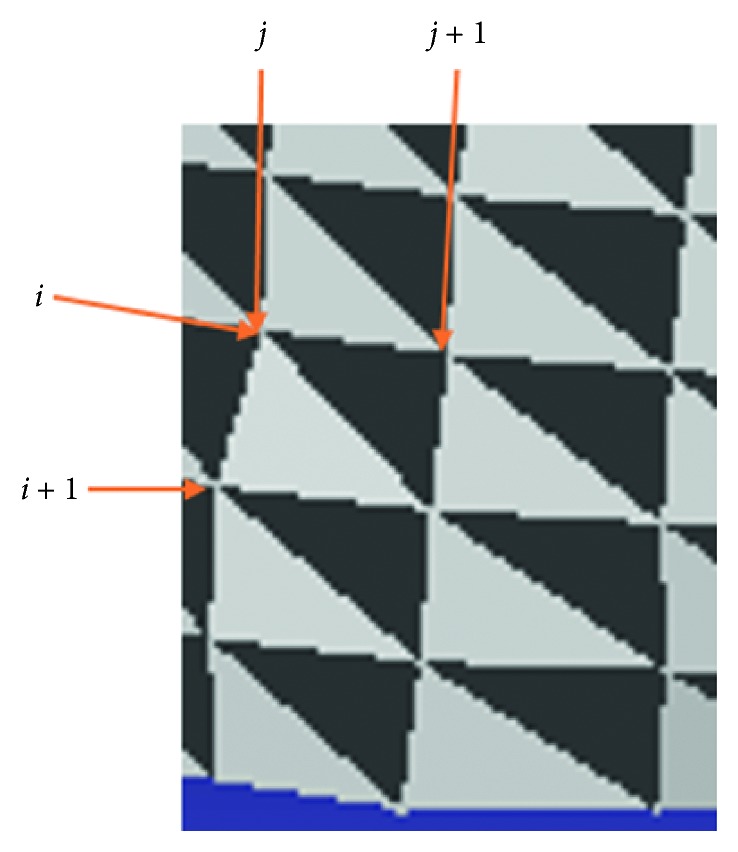
Remeshing based on triangular mesh.

Once all necessary slices are ready to build a 3D model, we perform remeshing counted on the triangular mesh method. This simple rule constitutes a mesh by using three points. The points at (*i*, *j*),  (*i*, *j*+1),  (*i*+1, *j*) on two adjacent slices would form a mesh. Likewise, the points at (*i*+1, *j*),  (*i*, *j*+1), (*i*+1, *j*+1) would also produces a mesh (Figure 16).

## 6. Discussion and Conclusion

Generating 3D models has been becoming an attractive field in recent years. There is no doubt about the versatility of the 3D model in computer graphics applications such as gaming, films, and garments. However, constructing a 3D shape is not a trivial task since the complexity of the model usually demands careful design, the power of computer hardware, and modern scanning devices. To tackle this problem, we introduced a novel method to create a new 3D model simply by taking the measurements as input. Our main contributions include (1) describing a formula to represent 3D data under slices of point cloud, (2) introducing two-step framework based on Neural Networks for generating the primary slices and impaling entire slices, and (3) conducting the experiment and unveiling a benchmark on the IUH and HUST 3D human dataset.

**Figure 17:**
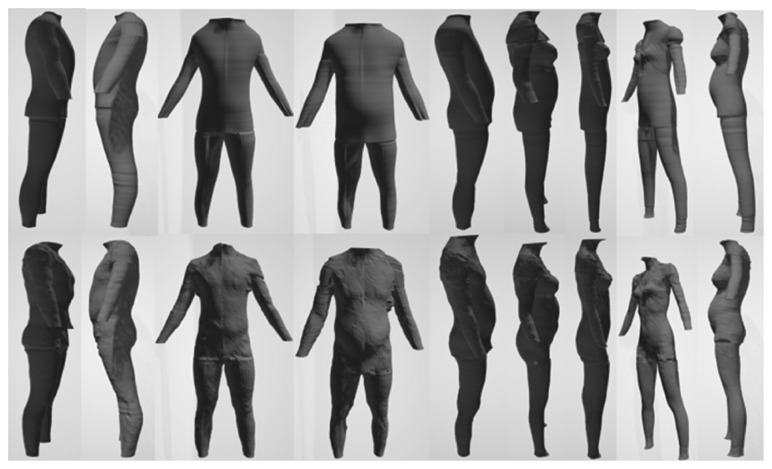
3D avatars of male and female. First row: generated shapes; second row: original shapes.

It is difficult to compare the present study's finding with other previous studies because of the different dataset and evaluating metrics. However, the results confirm the effectiveness of our approach because the generated 3D point cloud models are fine enough for visualization with the small error during the rational running time (Figure 17). Our proposed framework not only explores the correlation between the shape and the size of a human body but also captures the local information among adjacent slices. Instead of directly inferring a whole 3D model, we divided the objective model into specific parts and defined suitable NN architecture for each part. In the spirit of learning in detail slice shapes rather than learning overall structure, the hierarchical learning strategy was introduced in which the shapes of slices corresponding to user-defined measurements are the foundation of all other slices' shape.

The slice structure that we used in this study is not restricted in the static case. It is also effective when applying to 3D dynamic models via a morphable skeleton. The key idea to generate a new slice shape is to deform an initial shape depending on the training dataset. Because every single step of our method does not need to change the coordinate or reduce the dimension, we ensure that a generated point cloud still look like the samples in the training data. The main drawback of our approach is data deficiency. We suffer from the underfitting problem; hence, the NN systems cannot achieve the ideal generalization. The second weakness is that we concentrate on constructing point clouds, not meshes. Therefore, any application requiring 3D models with full mesh reconstruction might need more processing steps. Although slice structure is very simple, it is challenging to achieve its status, especially when disjointing 3D shapes with complex designs.

In conclusion, this study suggests that a 3D point cloud be constructed completely when giving a set of essential measurements. On the other hand, it is necessary to consider the shape in more detail when dealing with complicated 3D structures such as human bodies. Our proposed framework shed the light on this concern since it has the ability to analyze local shape features.

## Figures and Tables

**Algorithm 1:**
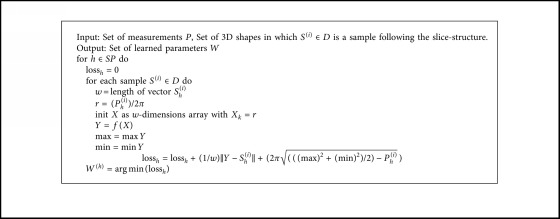
Building primary slices form measurements.

**Algorithm 2:**
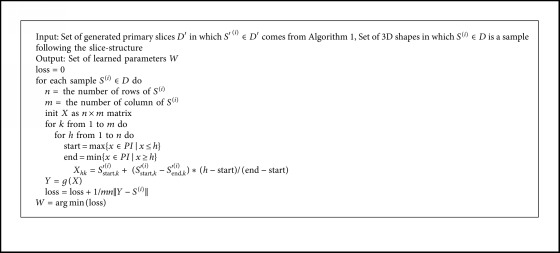
Constructing 3D point cloud.

**Table 1:** Summary of dataset.

Owner	Gender (age)	The amount of avatars	The amount of damaged avatars
Industrial University of Ho Chi Minh City (IUH)	Male (20–60)	1106	65
Hanoi University of Science and Technology (HUST)	Female (20–60)	600	63

**Table 2:** Average error on each principal slice on the training data of male and female datasets (full dataset).

Slice	Train/male (%)	Train/female (%)
Hip	8.39	4.68
Abdomen	4.41	3.46
Waist	5.34	4.90
Chest	5.80	11.25
Shoulder	6.18	9.37
Neck	13.55	21.61
Left wrist	7.77	10.99
Left forearm	5.53	7.48
Left elbow	4.21	7.72
Left biceps	6.22	8.80
Left overarm	10.54	15.66
Right wrist	13.43	8.12
Right forearm	12.69	7.63
Right elbow	8.36	7.01
Right biceps	8.84	6.69
Right overarm	12.20	12.64
Left ankle	7.93	7.38
Left calf	7.73	4.62
Left under knee	11.80	3.30
Left knee	6.31	4.40
Left middle thigh	3.63	4.72
Left thigh	6.46	9.67
Right ankle	9.54	6.64
Right calf	7.52	4.66
Right under knee	10.67	3.09
Right knee	5.80	4.45
Right middle thigh	3.41	5.05
Right thigh	6.03	10.21

**Table 3:** Average error on each part of 3D male and female models after activating the CNN model (the test set is comprised of damaged and undamaged samples).

Part	Train/male (%)	Train/female (%)	Test/male (undamaged) (%)	Test/female (undamaged) (%)	Test/male (damaged) (%)	Test/female (damaged) (%)	Test/male (average) (%)	Test/female (average) (%)
Torso	7.42	11.26	6.46	9.74	10.73	15.79	8.59	12.765
Left arm	8.24	15.20	6.68	15.83	10.87	26.11	8.77	20.97
Right arm	12.99	12.23	10.83	12.33	14.72	13.82	12.77	13.075
Left leg	8.78	7.26	7.59	7.40	11.22	9.92	9.40	8.66
Right leg	8.39	8.01	7.81	7.45	11.81	13.26	9.81	10.35
Average	**9.16**	**10.59**	**7.87**	**10.55**	**11.87**	**15.78**	**9.86**	**13.16**

**Table 4:** Average error on each part of 3D male and female models on the undamaged datasets before and after activating the CNN model.

	Train/male (before)	Train/female (before)	Test/male (before)	Test/female (before)	Train/male (after)	Train/female (after)	Test/male (after)	Test/female (after)
Torso	6.90	8.04	7.21	8.27	6.68	7.13	7.94	7.94
Left arm	7.62	12.77	8.31	13.81	7.15	12.60	7.67	13.83
Right arm	8.14	10.81	8.27	10.94	7.15	10.70	7.35	10.72
Left leg	7.02	6.32	7.74	6.51	7.31	5.77	7.90	5.96
Right leg	7.53	6.63	8.12	7.08	6.90	5.89	7.39	6.13
Average error	**7.42**	**8.91**	**7.93**	**9.32**	**7.03**	**8.41**	**7.65**	**8.91**

**Table 5:** Average training and testing time on both datasets.

Part	Train (s)	Test a sample (s)
Torso (primary × 6)	474	0.013
Torso (all)	1484	0.121
Arm (primary × 10)	540	0.032
Arm (all)	133	0.098
Leg (primary × 12)	780	0.035
Leg (all)	764	0.108
Total	**4175**	**0.407**

## Data Availability

The data used to support the findings of this study have not been made available because they are private.
