# Corrigendum to “Generating Point Cloud from Measurements and Shapes Based on Convolutional Neural Network: An Application for Building 3D Human Model”

**DOI:** 10.1155/2020/9126140

**Published:** 2020-06-28

**Authors:** Mau Tung Nguyen, Thanh Vu Dang, Minh Kieu Tran Thi, Pham The Bao

**Affiliations:** ^1^Hanoi University of Science and Technology, School of Textile—Leather and Fashion, Hanoi, Vietnam; ^2^Industrial University of Ho Chi Minh City, Ho Chi Minh City, Vietnam; ^3^Sai Gon University, Ho Chi Minh City, Vietnam

In the article titled “Generating Point Cloud from Measurements and Shapes Based on Convolutional Neural Network: An Application for Building 3D Human Model” [1], there was an error in affiliation number 1. In the corrected affiliation, “Hanoi” has been added to the institution name, and the city has been corrected from “Ho Chi Minh City” to “Hanoi.” The correct affiliation appears below.

Hanoi University of Science and Technology, School of Textile—Leather and Fashion, Hanoi, Vietnam.
